# Behavioral and physiological benefits of alpha-pinene in adult rats experiencing chronic stress: A focus on depression and oxidative stress

**DOI:** 10.1016/j.ibneur.2025.11.014

**Published:** 2025-11-24

**Authors:** Seyed Shahriyar Hosseini, Zohreh Ghotbeddin, Seyed Reza Fatemi Tabatabaei, Kaveh Rahimi

**Affiliations:** a Department of Basic Sciences, Faculty of Veterinary Medicine, Shahid Chamran University of Ahvaz, Ahvaz, Iran; b Stem Cell and Transgenic Technology Research Center, Shahid Chamran University of Ahvaz, Ahvaz, Iran

**Keywords:** Alpha-pinene, Maternal separation stress, Social behavioral, Depressive-like behavior, Corticosterone, Rat

## Abstract

To investigate the protective effects of alpha-pinene, an antioxidant compound, on depressive-like behaviors, social interactions, and biochemical markers in adult rats exposed to chronic stress through maternal separation during early development. Forty-nine Wistar rats were divided into seven groups: control (receiving solvent only), two alpha-pinene treatment groups (5 and 10 mg/kg), a stress group (maternal separation for 6 h daily for 21 days), two combined treatment groups (alpha-pinene with stress), and a positive control group (imipramine hydrochloride, 10 mg/kg). Behavioral assessments included forced swimming tests, sucrose preference tests, elevated plus maze tests, and social interaction tests. Serum corticosterone levels and oxidative stress markers (MDA and GPx activity) in brain tissue were measured. The stress group showed significant increases in immobility time, anxiety behaviors, and serum corticosterone levels, with decreased sucrose preference and altered social interactions. Biochemical analysis revealed increased MDA levels and decreased GPx activity in the stress group, indicating heightened oxidative stress. Alpha-pinene treatment at both doses significantly improved behavioral outcomes, reduced corticosterone levels, decreased MDA levels, and increased GPx activity in stressed rats, with effects comparable to imipramine. Alpha-pinene significantly protects against chronic stress-induced behavioral and biochemical alterations in rats. These findings suggest that alpha-pinene may improve behavioral performance through antioxidant mechanisms and by modulating HPA axis activity, as evidenced by reduced corticosterone levels. This study provides preliminary evidence for alpha-pinene's potential therapeutic value in stress-related disorders.

## 1 Introduction

Major Depressive Disorder (MDD) is a severe psychiatric illness that impacts millions of people globally and is frequently linked to prolonged exposure to stress (Otte et al., 2016). Early-life stress (ELS), especially during crucial developmental periods, is a recognized risk factor for the development of depressive-like behaviors in later life (Guzmán-Gutiérrez et al., 2015). A commonly utilized animal model to replicate ELS is maternal separation, which consistently produces behavioral and physiological alterations that mirror human depression (Ghotbeddin et al., 2020). Research shows that offspring subjected to maternal separation exhibit passive behaviors, which, in adulthood, are associated with heightened stress responses and neurodevelopmental disruptions, making them more susceptible to depression (Chen et al., 2025).

Among the biological processes associated with stress-induced depression, oxidative stress has attracted significant interest (Ghotbeddin et al., 2022). Prolonged stress increases the levels of reactive oxygen species (ROS) and weakens antioxidant defenses, resulting in neuronal injury and behavioral impairment. Biomarkers like malondialdehyde (MDA) and the activity of glutathione peroxidase (GPx) are frequently utilized to evaluate oxidative damage and antioxidant capacity in experimental models of depression (Ghotbeddin et al., 2024). Excessive reactive oxygen species (ROS) under oxidative stress (OS) conditions can disrupt normal physiological signaling, leading to the peroxidation of nucleic acids, lipids, and proteins, ultimately resulting in cellular damage and pathological responses (Ballard and Towarnicki, 2020). Specifically, the impairment of cell membrane lipid bilayers can result in abnormal transmembrane exchanges of various molecules and ions, thereby disturbing the physiological and biochemical homeostasis within the cell, which in turn can lead to cellular damage (Cobley et al., 2018).

Neurons, astrocytes, and microglia in the brain are abundant in mitochondria and NADPH oxidase (NOX), enabling them to produce significant amounts of ROS. Furthermore, the brain is an organ with high oxygen consumption, characterized by a large cortical surface area, a high concentration of unsaturated fatty acids, and a deficiency in antioxidant enzymes, which collectively contribute to a low antioxidant capacity and an increased susceptibility to oxidative stress (Ji et al., 2023).

Considering the central role of oxidative stress in the pathophysiology of depression, antioxidant agents have surfaced as potential therapeutic options. α-pinene, a naturally occurring monoterpene found in the oils of coniferous trees, has demonstrated significant antioxidant properties in multiple preclinical models.

Pinene (C₁₀H₁₆) is a bicyclic monoterpene hydrocarbon that features a double bond. Nature offers two isomers: α- and β-pinene, with α-pinene as the main component in the essential oils of coniferous trees. It is recognized for its strong antioxidant and anti-inflammatory properties, as well as its capability to neutralize free radicals (Liu et al., 2020).

Acikgoz et al. (2022) reported that α-pinene effectively prevents acute pancreatitis in rat models. Similarly, Pinheiro et al. demonstrated the protective gastrointestinal effects of α-pinene in experimental gastric ulcer models (Bekhbat et al., 2018). Furthermore, α-pinene has demonstrated significant anticancer activity against liver cancer and melanoma in rodent models (Sadeghi et al., 2016). Studies have shown that both α- and β-pinene have anxiolytic and antidepressant properties. For instance, intraperitoneal injection of β-pinene (100 mg/kg) increased locomotion and reduced anxiety in the forced swim test (Guzmán-Gutiérrez et al., 2015). Moreover, β-pinene increased locomotion by twofold compared to the antidepressant imipramine in the same (Guzmán-Gutiérrez et al., 2012).

While α-pinene also exhibits anti-inflammatory properties, this study primarily concentrates on its antioxidant efficacy in alleviating depressive-like behaviors triggered by maternal separation.

In pursuit of this objective, we examined the impact of α-pinene administration on behavioral performance, oxidative stress indicators (MDA and GPx), and serum corticosterone concentrations in adult male rats that experienced early-life maternal separation. This study seeks to elucidate the neuroprotective function of α-pinene by focusing on oxidative stress pathways within a chronic stress model of depression.

## 2 Methods

### 2.1 Animals and grouping

Forty-nine adult Wistar rats were randomly assigned to seven experimental groups (n = 7 per group):

1. Control group – received vehicle only, no stress exposure (stress-free baseline group). 2. Alpha-pinene 5 mg/kg group – received alpha-pinene without stress exposure (stress-free drug group) (Salehi et al., 2019). 3. Alpha-pinene 10 mg/kg group – received alpha-pinene without stress exposure (stress-free drug group) (Salehi et al., 2019). 4. Stress group – exposed to maternal separation stress (6 h/day for 21 consecutive days) without treatment. 5. Stress + alpha-pinene 5 mg/kg group – received alpha-pinene during stress exposure. 6. Stress + alpha-pinene 10 mg/kg group – received alpha-pinene during stress exposure. 7. Positive control group – received imipramine hydrochloride (10 mg/kg) during stress exposure (Guzmán-Gutiérrez et al., 2015).

Alpha-pinene dissolved in a suitable solvent; corn oil and it should be mentioned that all injections were done intraperitoneally.

Adult male and female Wistar rats were sourced from Shahid Chamran University of Ahvaz and maintained under standard laboratory conditions (22 ± 2°C, 12-hour light/dark cycle, lights activated at 7:00 AM) with unrestricted access to food and water. Following a period of acclimatization, breeding pairs were formed, and pregnant females were housed individually until they gave birth. The day of delivery was marked as postnatal day 0 (PND 0).

Maternal separation (MS) took place from PND 1 to PND 21. Pups designated for the MS groups were separated from their mothers for 6 h each day (from 9:00 AM to 3:00 PM). During this separation, the pups were placed in a temperature-controlled incubator (kept at 32–34°C) to avoid hypothermia and were housed in cages lined with clean bedding. The dams were returned to their home cages during the separation intervals. After each separation session, the pups were reunited with their mothers until the subsequent separation. Control pups that were not subjected to separation remained with their dams undisturbed, except for routine cage cleaning. All procedures were performed in compliance with institutional animal care guidelines and received approval from the appropriate ethics committee EE/1400.2.24.64245/scu.ac.ir.

### 2.2 Behavioral assessments

**Fig. 1:**
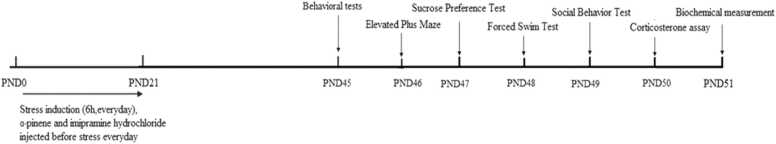
Timeline of the experiment.

Behavioral tests, conducted on adult male rats, included elevated plus maze, force swimming test, sucrose preference, and social behavior tests. The experimental timeline, including maternal separation protocol, alpha-pinene administration, and behavioral/biochemical assessments, is outlined in Fig. 1.

#### 2.2.1 Elevated plus-maze

The Elevated Plus Maze consists of four arms arranged in a "+ " shape, with two open arms and two enclosed arms. It is elevated above the ground to induce stress from the height, which increases anxiety in the animals. This test involves placing animals in the center of a maze and allowing them to freely explore for five minutes. Through a video tracking system, a camera is positioned above the maze and a camera is used to record their behavior. The time spent in open arms, the number of entries into open arms, the time spent in enclosed arms, and the number of entries into closed arms are key measurements. Observations of the animals' preference for open versus enclosed spaces provide a measure of anxiety-like behavior (Nazarizadeh et al., 2024)**.**

#### 2.2.2 Sucrose Preference Test (SPT)

The assessment of anhedonic behavior, which reflects depression-like conditions resulting from chronic stress, was conducted through the sucrose preference test. Prior to testing, each rat underwent a 23-hour period of food and water deprivation to heighten their motivational drive. After this deprivation, the animals were provided with simultaneous access to two bottles for a duration of one hour—one filled with tap water and the other with a 1 % sucrose solution. Although no formal adaptation phase was implemented, the design of the test permits rats to naturally sample and differentiate between the two liquids. Rodents exhibit a natural inclination towards sweet solutions, and this inherent preference facilitates the reliable identification of anhedonia without the need for prior training. To mitigate side bias, the positions of the bottles were counterbalanced among the animals. Fluid intake from each bottle was recorded both before and after the testing period, and the sucrose preference percentage (SPP) was computed using the following formula:

SPP = (Sucrose solution consumption / (Water consumption + Sucrose solution consumption)) × 100

During the course of the experiment, the body weight of each animal was measured weekly with a digital scale. This procedure aimed to evaluate the physiological effects of maternal separation stress and the possible modulatory influences of alpha-pinene and imipramine treatment. Body weight gain variations were examined across all groups, which included control, stress-exposed, treatment-only, and combined treatment groups. These measurements were taken into account to guarantee that fluctuations in body weight did not interfere with behavioral results, especially in the forced swimming test (Liu et al., 2020).

#### 2.2.3 Forced Swim Test (FST)

The Forced Swim Test was performed utilizing a clear cylindrical acrylic container with a diameter of 45 cm and a height of 79 cm. The container was filled with water to a depth of 60 cm, ensuring that the rat could not reach the bottom with its hind limbs or tail, thus encouraging swimming behavior and preventing any form of postural support. The water temperature was consistently maintained at 23–24 °C during the test to reduce thermal stress.

Each rat was carefully placed into the water, and its behavior was monitored for a duration of 5 min. Immobility was characterized by the cessation of active movements, with the animal remaining passively afloat in a vertical orientation. The length of immobility was manually recorded by trained observers who were unaware of the experimental conditions. This approach aligns with established protocols and facilitates a reliable evaluation of depression-like behavior (Ghotbeddin et al., 2020).

#### 2.2.4 Social behavior Test

The social interaction test was conducted in a 19 × 43 cm area divided into three parts with walls. Two wire chambers contained a stranger rat and a familiar rat. The test had three phases: In the habituation phase, the subject rat was placed in the central area for five minutes to get used to the environment, with exits blocked and empty chambers in the side compartments. In the first test phase, a stranger rat was introduced, and the rat could interact for 10 min. In the second test phase, a new stranger rat was added to assess the rat’s social preferences and relationships, also lasting 10 min (Acikgoz et al., 2022).

### 2.3 Biochemical measurements

#### 2.3.1 Hippocampal tissue sampling

At the end of behavioral tests, at least 4 rats from the selected groups were euthanized for hippocampal tissue sampling. Immediately after brain tissue removal, the brain was weighed. The hippocampus was dissected from both the left and right hemispheres using a scalpel, and the hippocampus was separated from the limbic system. After the samples were placed on ice compressors, they were transferred to a temperature of −70°C.

#### 2.3.2 Assessment of lipid peroxidation by measuring malondialdehyde levels

To evaluate lipid peroxidation, 50 μL of serum samples were mixed with 250 μL of a 20 % trichloroacetic acid solution and 100 μL of 0.6 % thiobarbituric acid. A boiling water bath was used to heat the mixture for at least 20 min. A centrifuge was used to remove impurities and clarify the supernatant after cooling the samples. Following that, 200 μL of the supernatant was transferred to a 96-well plate, and an absorbance at 535 nm was measured using a spectrophotometer against a blank (200 liters of distilled water instead of the sample). The quantity of MDA was determined through the equation C = OD/1.56 × 10^5, with results reported in micromoles per liter (Bio-Tek, Winooski, VT, USA) (Ghotbeddin et al., 2022)**.**

#### 2.3.3 Glutathione peroxidase (GPx) activity assay

The activity of GPx in the hippocampal tissue was determined using a colorimetric enzymatic assay kit (Sigma-Aldrich, USA). Tissue samples were homogenized on ice and centrifuged, and the supernatant was used for analysis. The assay was performed according to the manufacturer’s instructions. In this method, GPx catalyzes the reduction of hydrogen peroxide in the presence of reduced glutathione. The oxidized glutathione is subsequently converted back to its reduced form by glutathione reductase, using NADPH as a cofactor. The oxidation of NADPH to NADP⁺ results in a gradual decrease in absorbance, which was recorded at 340 nm using a microplate reader. Enzyme activity was calculated from the rate of NADPH consumption and expressed as international units per milligram of protein (IU/mg) (Ghotbeddin et al., 2024).

### 2.4 Corticosterone measurement

Following the behavioral tests, the rats were anesthetized with carbon dioxide, and blood samples were collected from their hearts. For serum separation, blood samples were quickly collected into tubes and centrifuged at 3500 rpm for 10 min. Afterwards, cortisol hormone concentration was measured using the Immunotech assay kit (Bekhbat et al., 2018).

### 2.5 Statistical analysis

All data are presented as mean ± standard error of the mean (Mean ± SEM). GraphPad Prism 9 software was used for statistical analysis and graphing, and (p < 0.05) was set as the significance level. Various parameters were compared between experimental groups after testing for normality. In addition to normality testing, one-way ANOVA and Tukey's post-hoc tests were performed.

## 3 Results

### 3.1 Behavioral outcomes

#### 3.1.1 Depressive-like behaviors results

**Fig. 2:**
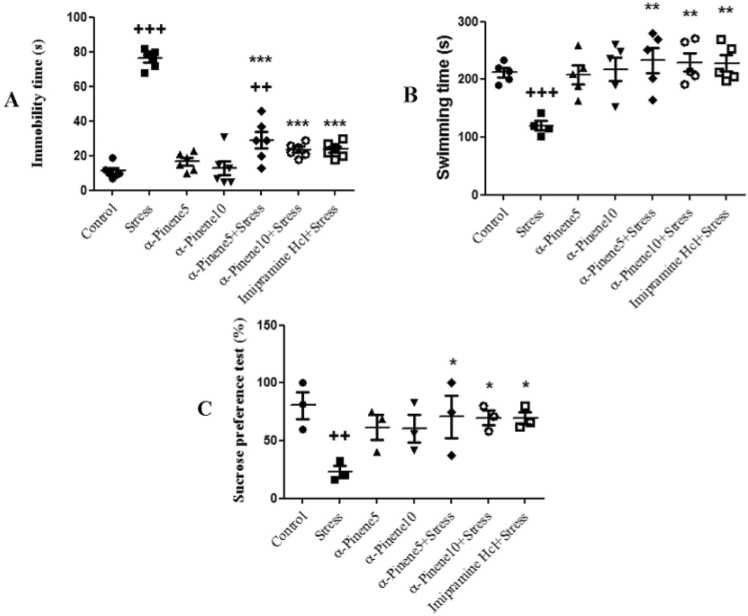
Comparison of the mean immobility (A) and swimming time (B) in the forced swim test and of the mean sucrose preference percentage (C) between experimental groups. + +, and + ++ indicate significant differences at (p < 0.01) and (p < 0.001), respectively, between the experimental groups and the control group. ***, ** and * indicate significant difference at (p < 0.001), (p < 0.01) and (p < 0.05) between the drug + stress groups and the stress group. Data are presented as mean ± SEM (n = 7).

The figures of Forced Swim Test and Sucrose Preference Test have been combined into a single figure to represent depressive-like behaviors (Fig. 2). The highest level of inactivity was observed in the stress group, which was separated from their mothers for 6 h every day. According to the results of one-way ANOVA, the inactivity time in the stress group (p < 0.001) and the alpha-pinene 5 + stress group (p < 0.01) showed a significant increase compared to the control group. The use of alpha-pinene 5 and 10 during the stress application significantly reduced inactivity time in these groups compared to the stress group (p < 0.001). Inactivity time in the imipramine + stress group also significantly decreased compared to the stress group (p < 0.001) (Fig. 2A). As a result of being separated from their mothers for six hours daily, the stress group had the shortest swimming time. In the stress group, the mean swimming duration significantly decreased compared to the control group (p < 0.001). Compared to the stress group, the alpha-pinene 5, 10, and imipramine-treated groups significantly increased their swimming time compared to the stress group (p < 0.01) (Fig. 2B).

The lowest percentage of sucrose consumption was observed in the stress group, while the highest amount was consumed by the control group, which had not been subjected to any stress. The results indicated a significant difference between the stress group and the control group, with sucrose consumption in the stress group showing a significant decrease compared to the control group (p < 0.01). Sucrose consumption in the stress groups that received alpha-pinene at doses of 5 mg/kg, 10 mg/kg, and imipramine hydrochloride was significantly increased compared to the stress-only group (p < 0.01) (Fig. 2C).

#### 3.1.2 Results of anxiety-like behaviors

**Fig. 3:**
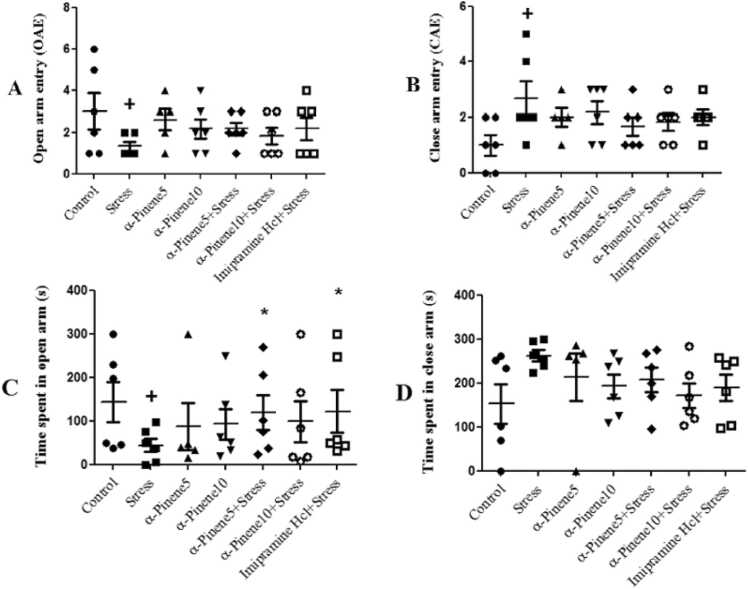
Comparison of the mean number of entries into the open (A) and close arms (B) and the mean time spent in the open (C) and close arms (D) in the Elevated Plus-Maze Test. + indicates a significant difference at the (p < 0.05) level between the stress group and the control group. * present a significant difference at the (p < 0.05) level between the drug + stress groups and the stress-only group. Data are presented as mean ± SEM (n = 7).

The highest number of entries into the open arm was observed in the control group, while the lowest number was found in the stress group. A significant difference was found only between the stress group and the control group in the number of entries into the open arm, with the stress group showing a significant decrease in the number of entries compared to the control group (p < 0.05) (Fig. 3A). Among the experimental groups, the highest number of entries into the closed arm was observed in the stress group, while the lowest number was found in the control group. The number of entries into the closed arm in the stress group showed a significant increase compared to the control group (p < 0.05) (Fig. 3B). The open-arm time spent by the stress group was significantly shorter than that of the control group. Compared to the stress-only group, those who received alpha-pinene at 5 mg/kg and imipramine spent more time in the open arm (p < 0.05) (Fig. 3C). No significant difference was observed in the mean time spent in the closed arm between the experimental groups (Fig. 3D).

#### 3.1.3 Results of the social behavior assessment

**Fig. 4:**
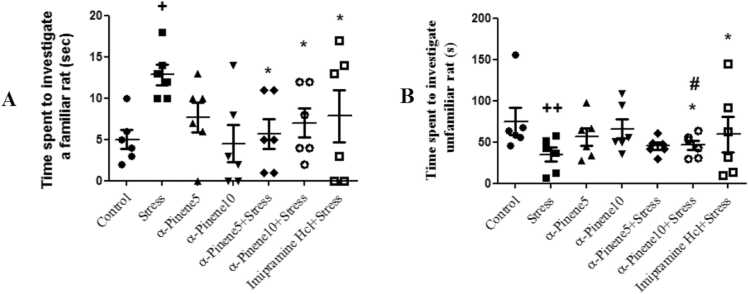
Comparison of the mean time spent around the familiar (A) and unfamiliar rats (B) in the Social Behavior Assessment test. + and + + indicate significant differences between the stress and control groups at the (p < 0.05) and (p < 0.01) levels. * Indicates a significant difference at the (p < 0.05) level between the drug + stress groups and the stress-only group. # shows a significant difference between the alpha-pinene 10 + stress group and the alpha-pinene 10-only group (p < 0.05). Data are presented as mean ± SEM (n = 7).

According to the study, the stress group spent more time around the familiar rat than the control group (p < 0.05). There was no significant difference between the other groups and the control group. Compared to the stress-only group, time spent around the familiar rat was significantly reduced in the stress groups receiving alpha-pinene at 5 mg/kg, 10 mg/kg, and imipramine hydrochloride (Fig. 4A).

A significant reduction in time spent around the unfamiliar mouse was observed in the stress group compared to the control group (p < 0.05). The time spent around the unfamiliar mouse increased significantly in the alpha-pinene 10 + stress and imipramine hydrochloride + stress groups (p < 0.05). Furthermore, this time was significantly decreased in the alpha-pinene 10 + stress group when compared to the alpha-pinene 10 only group (p < 0.05) (Fig. 4B).

### 3.2 Biochemical outcomes

#### 3.2.1 Results of serum corticosterone levels and oxidative stress in brain tissue

**Fig. 5:**
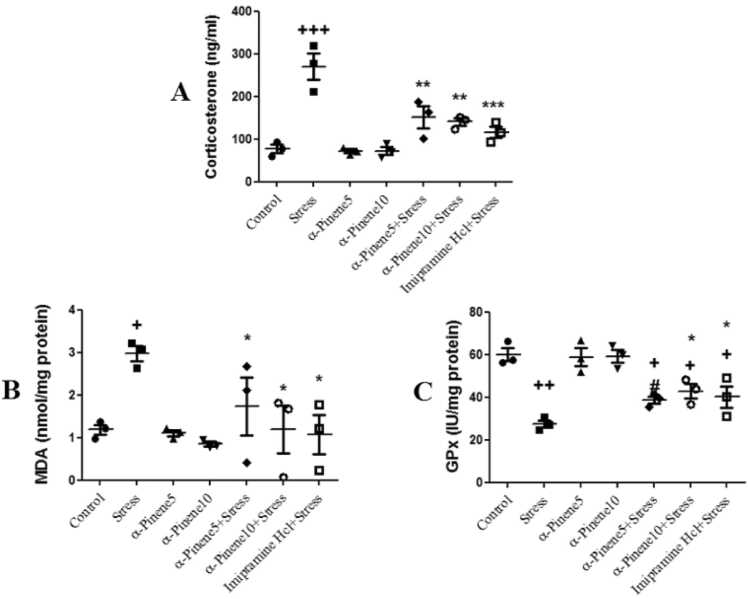
Comparison of the mean serum corticosterone levels (A), malondialdehyde (MDA) (B) and glutathione peroxidase (GPx) (C) levels in brain tissue between the study groups. + , + + and + ++ indicate a significant differences between the stress and control groups respectively at the (p < 0.05) (p < 0.01) (p < 0.001) levels. *, ** and *** show significant differences at the (p < 0.05), (p < 0.01) and (p < 0.001) levels, between the drug + stress groups and the stress-only group. Data are presented as mean ± SEM (n = 4).

The stress group had the highest serum corticosterone levels. As compared to the control group, the stress group showed significantly higher levels of serum corticosterone (p < 0.001). Fig. 5A shows that serum corticosterone levels were significantly lower in the alpha-pinene + stress group (p < 0.01) and the imipramine hydrochloride + stress group (p < 0.01).

Stress-induced increases in MDA levels were significant (p < 0.05) compared to the control group. Compared to the stress-only group, drug + stress groups had significantly lower MDA levels (p < 0.05) (Fig. 5B).

The GPx levels in the stress group (p < 0.05), alpha-pinene 5 + stress (p < 0.05), alpha-pinene 10 + stress (p < 0.05), and imipramine + stress group were significantly decreased compared to the control group (p < 0.05). GPx levels in the alpha-pinene 10 + stress and imipramine + stress groups were significantly increased compared to the stress-only group (p < 0.05) (Fig. 5C).

## 4 Discussion

Studies have shown that chronic stress can induce depression-like behaviors in rodents (Sadeghi et al., 2016). In the present study, chronic stress also led to the development of depression-like behaviors in the rats that were separated from their mother from PND0 to PND21.

Chronic and prolonged stress is one of the most important and primary causes of depression, leading to the involvement of brain regions such as the hippocampus, amygdala, anterior cingulate cortex, and the cerebral cortex. Previous research has shown that hippocampal atrophy occurs in association with depression, and the hippocampus plays a crucial role in the pathophysiology of depression (Rao et al., 2010).

According to the monoamine hypothesis proposed by researchers in the early 1950s, depression is caused by a deficiency of monoamines, particularly norepinephrine and serotonin. In fact, depression results from the deficiency of monoamines, including norepinephrine, dopamine, and serotonin (Madrigal et al., 2001).

Recent studies have shown that early life stress-inducing events affect the hippocampus and the prefrontal cortex, thalamus, and hypothalamus. In response to stress, the hypothalamus, pituitary, and adrenal (HPA) axis play an important role. The hypothalamus modulates nervous, endocrine, and autonomic systems. Some studies have shown that stress can cause depression-like behaviors in rodents (Sadeghi et al., 2016), which is consistent with our findings. One of the depression-like behaviors in rodents is an increase in immobility time in the forced swim test (Aksu et al., 2009). The activity of the HPA axis is tightly regulated by GABAergic signaling; in fact, the deficiency of neurosteroids and GABA in depressed patients indicates the role of GABAergic signaling in depression (Maguire, 2019).

A high level of serum corticosterone was observed in the stress group. Compared to the control group, the stress group showed significantly higher serum corticosterone levels. Safari et al. (2020) examined the effects of swimming exercise on corticosterone levels in adult male Wistar rats. Their results showed that the group that underwent swimming exercise had lower corticosterone levels than the stress-only group (Safari et al., 2020). Physical and psychological stress increases corticosterone primarily through hypothalamic-pituitary-adrenal (HPA) axis activity. Stress causes continuous cortisol production, resulting in neurodegeneration, inflammation, hippocampal volume reduction, anxiety, and depression-like behavior (Andersen et al., 2013).

Increasing levels of biomarkers of oxidative stress are associated with depression, according to a meta-analysis study. However, depression's development remains largely misunderstood (Black et al., 2015). It has been shown that oxidative stress, inflammatory processes, and depressive disorders are directly related. ROS secretion increases under stressful conditions, which results in increased oxidative stress, reduced ATP levels, and glycolysis inhibition. Furthermore, glutathione, which is a potent antioxidant, is also reduced (Herbet et al., 2018). The sympathetic nervous system and the HPA axis are activated by almost all stressors. ACTH is released when the HPA axis is activated, which increases cortisol secretion. The consequence of this is the onset of inflammation and oxidative stress.

Alpha-pinene exhibits antioxidant properties according to the findings of the present study. In the alpha-pinene-treated groups, MDA levels were lower and GPx activity was higher than in the stress-treated groups. Bagheri et al. showed that alpha-pinene reduces oxidative stress in diabetic mice in their study (Bagheri et al., 2019). A study by Hashemi et al. (2023) investigated alpha-pinene's effects on spatial and working memory in rats. In comparison to a control group, alpha-pinene reduced malondialdehyde levels in hippocampal tissue at a dose of 10 mg/kg. In addition, alpha-pinene improved rats' spatial performance and working memory.

This research was carried out solely on male rats to minimize biological variability and to create a fundamental understanding of the behavioral and neurobiological effects of early-life stress. Although this method facilitated a more controlled analysis of the results, we recognize that the omission of female subjects restricts the applicability of our findings. Future research is intended to incorporate both male and female rats to explore possible sex-specific variations in vulnerability to depression-like and anxiety-like behaviors, in line with NIH guidelines and the increasing acknowledgment of sex as a biological variable in preclinical studies.

## 5 Conclusions

In summary, our results showed that chronic stress caused by maternal separation during the critical early life stages led to increased anxiety and depression-like behaviors, decreased social interactions, increased serum corticosterone levels, and enhanced oxidative stress. The stress group, however, had reduced anxiety and depression, improved social interactions, lower corticosterone levels, and reduced oxidative stress after treatment with alpha-pinene (5 and 10 mg/kg). Alpha-pinene doses were not significantly different between the two trials. According to the results, alpha-pinene reduces oxidative stress in rats that were separated from their mothers during the critical early postnatal days by improving depression-like behavioral traits and social behavior.

## CRediT authorship contribution statement

**Zohreh Ghotbeddin:** Writing – review & editing, Supervision, Funding acquisition, Conceptualization.

## Ethics approval (research involving animals)

Animal studies were conducted according to the protocols and guidelines approved by the Institutional Ethics Committee of Shahid Chamran University of Ahvaz (EE/1400.2.24.64245/scu.ac.ir) and were conducted under the Guide for the Care and Use of Laboratory Animals (NIH).

## Funding

We are grateful to the Research Council of the 10.13039/501100005412Shahid Chamran University of Ahvaz for financial support.

## Declaration of Competing Interest

The authors declare no conflict of interest.

## Data Availability

The datasets generated for this study are available on request to the corresponding author.
